# An assessment of schoolyard renovation strategies to encourage children's physical activity

**DOI:** 10.1186/1479-5868-8-27

**Published:** 2011-04-09

**Authors:** Peter Anthamatten, Lois Brink, Sarah Lampe, Emily Greenwood, Beverly Kingston, Claudio Nigg

**Affiliations:** 1 Department of Geography and Environmental Sciences, University of Colorado Denver, Denver, CO, USA; 2 Department of Landscape Architecture, University of Colorado Denver, Denver, CO, USA; 3 Colorado Center for Community Development, College of Architecture and Planning, University of Colorado Denver, Denver, CO, USA; 4 Adams County Youth Initiative, Denver, CO, USA; 5 Department of Public Health Sciences, University of Hawaii at Manoa, Honolulu, HI, USA

## Abstract

**Background:**

Children in poor and minority neighborhoods often lack adequate environmental support for healthy physical development and community interventions designed to improve physical activity resources serve as an important approach to addressing obesity. In Denver, the Learning Landscapes (LL) program has constructed over 98 culturally-tailored schoolyard play spaces at elementary schools with the goal to encourage utilization of play spaces and physical activity. In spite of enthusiasm about such projects to improve urban environments, little work has evaluated their impact or success in achieving their stated objectives. This study evaluates the impacts of LL construction and recency of renovation on schoolyard utilization and the physical activity rates of children, both during and outside of school, using an observational study design.

**Methods:**

This study employs a quantitative method for evaluating levels of physical activity of individuals and associated environmental characteristics in play and leisure environments. Schools were selected on the basis of their participation in the LL program, the recency of schoolyard renovation, the size of the school, and the social and demographic characteristics of the school population. Activity in the schoolyards was measured using the System for Observing Play and Leisure Activity (SOPLAY), a validated quantitative method for evaluating levels of physical activity of individuals in play and leisure environments. Trained observers collected measurements before school, during school recess, after school, and on weekends. Overall utilization (the total number of children observed on the grounds) and the rate of activity (the percentage of children observed who were physically active) were analyzed. Observations were compared using t-tests and the data were stratified by gender for further analysis. In order to assess the impacts of LL renovation, recently-constructed LL schoolyards were compared to LL schoolyards with older construction, as well as un-renovated schoolyards.

**Results:**

Overall utilization was significantly higher at LL schools than at un-renovated schools for most observation periods. Notably, LL renovation had no impact on girl's utilization on the weekends, although differences were observed for all other periods. There were no differences in rates of activity for any comparison. With the exception of the number of boys observed, there was no statistically significant difference in activity when recently-constructed LL schools are compared to LL schools with older construction dates and there was no difference observed in comparisons of older LL with unrenovated sites.

**Conclusions:**

While we observed greater utilization and physical activity in schools with LL, the impact of specific features of LL renovation is not clear. However, schoolyard renovation and programs to encourage schoolyard use before and after school may offer a means to encourage greater physical activity among children, and girls in particular. Additional study of schoolyard renovation may shed light on the specific reasons for these findings or suggest effective policies to improve the physical activity resources of poor and minority neighborhoods.

## Background

Obesity has become an increasingly troublesome health problem in both wealthy and poor regions around the world [1]; the World Health Organization reports that there are now over one billion overweight adults, at least 300 million of whom are obese [2]. Obesity presents a particularly alarming health concern in the United States, where recent estimates are that approximately one third of children in the United States are considered overweight or obese [3].

Policy designed to change obesogenic environments aims to implement change that both reduce energy intake (by encouraging a healthy diet) and provide opportunities for increased energy output (by encouraging physical activity) [4]. Although behavioral change is a critical component to addressing obesity, interventions designed to modify individual behavior to reduce caloric intake have had limited success in preventing obesity on a long-term basis [3]. Although there is disagreement about which side of the energy equation is the most effective in terms of policy, extensive research has demonstrated that the built environment plays a key role in obesity-related behavior (e.g., [5-8]).

While there may be only a weak to moderate link between physical activity and obesity rates, there are numerous additional health benefits associated with increased physical activity. A recent literature review reports that physical activity is linked with reduced blood pressure, lower levels of cholesterol and blood lipids, reduced incidence of metabolic syndrome, increased bone mineral density, as well as reduced rates of depression [9]. Understanding the relationship between the built environment and physical activity and the specific implications of particular modifications to the built environment may contribute to strategies to reduce obesity prevalence [10] in addition to these other health benefits.

Along with transportation patterns and land-use patterns, design features are one of the key areas of inquiry in studies of the built environment and physical activity [11-13]. A number of specific design features pertaining to children's environments have been investigated in previous research. Time spent outdoors, access to recreational facilities and schoolyards [8,14] and proximity and number of play spaces and facilities to home [15] are associated with higher levels of physical activity in children and adolescents. Adding an additional recess period each day is associated with greater physical activity [16], while limited outdoor play time has been found to correlate with a high body mass index in young children [17]. If it is designed well, the outdoor built environment can create opportunities for healthy behavior change among children.

Compared to white children, children from African-American and Hispanic ethnicities are particularly likely to suffer from obesity, experience abnormally high glucose levels, and suffer from a higher prevalence of diabetes [18-20]. These observations could be attributed in part to the social and built environments of many minority children living in impoverished urban neighborhoods, which often fail to support healthy development and provide limited opportunities for healthy behavior, especially with respect to physical activity. Poor and minority children often have limited access to outdoor play spaces and structured opportunities for involvement in organized sports and other activities [19] and are more likely to have lower fitness levels. The reasons behind these disparities are nuanced and complex, but it might be possible to develop planning and design policies to address geographic inequality. Some research has shown that parents in low-income neighbourhoods have increasingly restricted their children's activity out of concerns for safety [21], and that design policies can have an impact on this concern [22]. Interventions to improve the safety of schoolyards have been shown to improve schoolyard utilization [23]. Indeed, communities that are designed to support physical activity have been found to have 100% higher rates of sufficient physical activity than those with no supportive attributes [8]. Facets of the built environment, such as the density of residences [24], general walkability [25], and the availability of recreational spaces and facilities [26,27] may be also linked to physical activity, although research often yields mixed results [21].

With the average child spending 1300 hours at school each year, schools are a valuable physical environment and social resource in efforts to promote physical activity. School wellness policies, community building initiatives, and walk-to-school programs [21] are a few examples of school-based strategies designed to encourage physical activity. In spite of a flourishing body of social-scientific and public health literature examining the relationship between physical activity and urban environments, there remains little work that specifically examines the impacts of recreational space renovation at schools. There is evidence that renovated schoolyards are more widely used by adults and children (especially boys) than un-renovated schoolyards [28]. Small, inexpensive interventions that have changed the structure of the physical environment, especially within the school environment, have shown significant positive correlations with physical activity levels. These interventions include painting the schoolyards [29], providing game equipment [30], and even increasing the number of balls available to youth [31]. Higher physical activity levels have been observed in schoolyards that have multicolored painting compared to those without [32]. The incorporation of culturally-tailored schoolyard elements may also encourage physical activity and ultimately contribute to the reduction of obesity [33], which is especially important in communities with large ethnic minority populations. In a study that used the same observation method as the current one, Sallis et al. conclude that making improvements to school environments could increase the physical activity of students throughout the school day [34]. After observing physical activity across twenty-four middle schools in San Diego, they determined that physical amenities, such as area type, area size, and permanent improvements (such as basketball courts and football goals) were associated with increased physical activity among both boys and girls. The authors conclude that "if we build it, they will come" [34]; that school design and renovation efforts may improve physical activity among children.

### Learning Landscapes

A Learning Landscape (LL) is a novel type of schoolyard that offers a diversity of elements lacking in traditional schoolyards. Such elements include schoolyard gateways, shade structures, banners, gardens, public art, student art, and art tile projects. LLs are designed and built by a non-profit partnership between the University of Colorado Denver's College of Architecture and Planning and a local, urban school district. Since its inception, LLs has attracted the involvement of 8,000 community volunteers, 18,000 students, 250,000 community members, 250 Americorps volunteers, and 20 volunteer organizations. The initiative has raised 47 million dollars for the completion of 82 new LL schoolyard sites.

After six years of collaboration between parents, elementary school students, staff, faculty, neighbors, local businesses and landscape architecture graduate students, the first schoolyard was completed in 1998. Although the project merely constructed redesigned schoolyards initially, it has since evolved into a city-wide initiative that redefines traditional school grounds and opens them up for community use outside of school hours. LL projects fulfilled a fundamental goal of landscape architecture: "to engage in scholarly activities that strike a balance between traditional academic and professional endeavors, while at the same time stretching the boundaries of landscape architecture design" [35].

The LL Initiative has transformed 82 neglected Denver elementary school schoolyards into attractive and safe multi-use schoolyards that are tailored to the needs and desires of the local community. This program has been sponsored by a broad-based, public-private partnership and is directed by expert faculty and masters-level students from the Department of Landscape Architecture at the University of Colorado at Denver. In 2000, Brink's UC Denver Program partnered with a local school district and private foundations to raise funds to construct 22 inner-city schoolyards. Since that time, local bonds have been passed to secure funding for additional LLs for a total of 98 by the end of 2012.

Success of LLs has traditionally been evaluated through a collaborative effort between LLs, the school district, students, community leaders, and city officials, using pre- and post-construction surveys and focus groups. The extent of this program produces a valuable opportunity to assess the impacts of schoolyard renovation on children's physical activity patterns. The goal of this study is to investigate the effect of these schoolyard renovations in low-income urban areas on physical activity among children by comparing utilization of LL schoolyards with matched control schoolyards. Specifically, we wish to consider 1) whether LL schoolyards are utilized more than non-LL schoolyards and 2) whether children utilizing LL schoolyards are more likely to exhibit moderate- to vigorous physical activity behaviour. A secondary objective is to evaluate whether the recency of schoolyard renovation has an impact on utilization and physical activity.

## Methods

Owing to the fact that the local school district selected which schools participated in the LL program, randomization was not possible. Constructed LLs were matched with schools possessing recently-built LLs (constructed within the past year), as well as control sites lacking schoolyard renovation. Case selection criteria included the percentage of students receiving free or reduced lunch, students' race and ethnicity, and school size, based on the total number of students enrolled in the school (table 1). After permission was obtained from the school district, principles of candidate schools were approached and permission was requested to conduct the study on their grounds. All the schools that were approached agreed to participate in the study. The study was approved by the Colorado Multiple Institutional Review Board.

**Table 1:** Characteristics of Study and Control Schools

			Ethnicity			
	School Enrollment	Free & Reduced Lunch	*African American*	*Latino*	*Anglo*	*Asian*
**Group A**						
**Renovated**	336	94%	56%	41%	2%	1%
**Recently rennovated**	219	88%	72%	26%	1%	1%
**Control**	272	91%	66%	30%	2%	2%
**Total**	**827**	**91%**	**65%**	**32%**	**2%**	**1%**
**Group B**						
**Renovated**	605	97%	-	94%	3%	3%
**Recently rennovated**	492	96%	6%	88%	3%	2%
**Control**	579	92%	-	94%	4%	2%
**Total**	**1676**	**95%**	**2%**	**92%**	**3%**	**2%**
**Group C**						
**Renovated**	350	94%	2%	88%	8%	2%
**Recently rennovated**	385	90%	6%	76%	10%	8%
**Control**	450	94%	4%	91%	4%	1%
**Total**	**1185**	**93%**	**4%**	**85%**	**7%**	**3%**

Because inclusion in the LL program was initially provided for low-income schools, the schools eligible for this study were located in deprived neighborhoods, populated largely by ethnic minorities. Study schools are therefore located in Denver neighborhoods facing significant social, economic, and educational challenges. Study sites were chosen from three different locations in Denver. Group A schools are from a predominately African-American neighborhoods characterized by significant gang activity. Group B and C schools are in neighborhoods located between three and five miles from downtown Denver and are comprised of poor, predominately Latino neighbourhoods. Schools in groups B and C are similar with respect to income and ethnicity, but are distinguished by school size (group B schools have an average attendance of 559 and group C schools an average attendance of 395). These groups were formed in order to select adequate controls on the basis of income, ethnicity, and school size. Each of the groups contains one school that had LL construction within a year before the data were collected, one school with older LL construction (constructed two or more years prior to the newly-renovated schoolyards) and one school without renovated school grounds (i.e., with no LL construction). Children observed in this study were elementary school students, between six and eleven years old.

Children were observed using the System for Observing Play and Leisure Activity in Youth (SOPLAY). SOPLAY is a quantitative method for evaluating levels of physical activity of individuals and associated environmental characteristics in play and leisure environment [36]. Target areas are predetermined and defined as "locations likely to provide opportunities for students to be physically active" [37] in which observers record the number of individuals present, their activity levels, and their gender. Each school yard was divided into activity zones that expressed the ground plane condition and the type of activity occurring. The observation protocol requires the identification of schoolyard variables with the greatest impact on children's physical activity based on area type, size, and permanent improvements. Observers were trained in SOPLAY observation methodology by a certified SOPLAY instructor. Children who are observed to be sedentary or walking are not considered to be physically active and children who are observed engaging in vigorous physical activity or in a "primary activity" such as using the schoolyard equipment such as a swing or jungle gym, are considered to be physically active. While the observers are able to distinguish between children and adults, they were not able to account for the age of specific children.

Observations were conducted over four days at each school to obtain accurate measurements. Two observers simultaneously observed the activity area for 20% of the total data collection time to test the reliability of the data, resulting in a reliability estimate of 87%. Observers were not part of the research team to ensure accuracy of the data collected. Data were collected from schoolyards at participating elementary schools in Denver between September 19 and October 29, 2005 and between September 29 and October 19, 2006 at regular time periods before the beginning of school, during school recess, after the school finished, and during the weekends. Because the observations covered the entire schoolyards, total schoolyard use and the number of children on the schoolyard at any particular observation point could be estimated from these surveys. Observational scans capturing activity on the entire schoolyard were treated as the unit of analysis. Between 28 and 30 schoolyard observations were conducted at each site.

### Study Design

While utilization of the schoolyards is more or less mandatory for the children during school recess, children may optionally use the schoolyard facilities before or after school or on weekends. It is hypothesized that by building innovative, culturally-sensitive schoolyard facilities, more children will be attracted to using the schoolyard facilities, particularly during these optional periods. In order to address this question, the number of children observed utilizing the schoolyards before school, during lunch recess, after school, and on weekends is compared across LL and non-LL schools in t-tests. Additionally, the data were stratified by gender to examine whether there were gender-based differences in utilization. In order to account for the enrollment differences within the study groups, all observations are standardized against total school enrollment and figures are reported as the number of children observed per 100 children enrolled (children attending the school). Although children attending the school may utilize schoolyards, particularly on weekends, schoolyard use is predominately by children from the school. Because the intent of this study is to evaluate schoolyards as means of encouraging school yard utilization and physical activity among children, two different outcome measures are reported: 1) the number of children observed on the playground and 2) the percentage of children engaged in moderate to vigorous physical activity. The first measure is intended to evaluate the impacts of LL renovation on schoolyard *utilization*, while the second is intended to estimate *how *schoolyards are utilized with respect to physical activity.

Some previous work has indicated that renovation of park features, such as trails [38] and playgrounds [28], results in greater utilization. In order to examine the specific impacts of LLs--independent of whether construction occurred recently--we repeated the same series of t-tests, but compared LL schoolyards built within a year prior to the study with those built two or more years prior, and also separately compared both groups of LL (recently and not-recently constructed) schoolyards to the unrenovated controls. Stratifying the sites this way allowed us to evaluate whether utilization is associated with the recency of construction, if differences persist among older LL schoolyards, or perhaps if observed differences are due instead to some combination of the two factors. Due to the even greater loss of statistical power resulting from additional stratification, schools are not stratified into separate observational periods for this final analysis.

## Results

Total schoolyard use was compared between LL and non-LL schools (where our assumed null hypothesis is *H*_*0 *_*: μ*_*1 *_*= μ*_*2*_). When all periods are taken into consideration, there was significantly greater utilization of LL schoolyards (table 2). Reported as the number of children per 100 children enrolled at the school, an average of 14.5 children was observed on LL schoolyards compared to 9.8 children in non-renovated schoolyards. Significance is achieved among all categories, except for boys before and after school and girls on weekends. Different observation periods yielded contrasting utilization patterns. The disparity between use of the study and control schoolyards was particularly large during lunch recess. While boys generally displayed somewhat greater utilization overall, the difference observed between renovated and unrenovated schoolyards was similar for boys and girls across all periods.

**Table 2:** Comparison of all children observed on schoolyards at LL and control schools during different utilization periods, reported as number of children per 100 children enrolled in the school

	Mean usage, Learning Landscapes	Mean usage, unrenovated schoolyards	Mean difference	Standard error difference	p
**All periods**					
All children	14.5	9.8	4.6	1.7	**.008
Boys	7.7	5.3	2.4	1.1	*.023
Girls	6.8	4.5	2.3	0.8	**.008
**Before School**					
All children	22.3	10.8	11.5	6.0	.066
Boys	12.7	6.4	6.3	3.7	.106
Girls	9.6	4.4	5.3	2.4	*.040
**Lunch Recess**					
All children	27.4	20.8	6.6	2.4	**.007
Boys	14.4	10.9	2.8	1.3	**.007
Girls	13.0	9.9	3.1	1.3	*.016
**After School**					
All children	4.2	2.1	2.1	0.8	*.007
Boys	2.0	1.2	0.8	0.4	.065
Girls	2.2	0.8	1.3	0.3	**.001
**Weekends**					
All children	0.8	0.2	0.6	0.3	*.068
Boys	0.5	0.1	0.4	0.2	*.025
Girls	0.2	0.1	0.2	0.1	.083

* p < .05 ** p < .01

As anticipated, the greatest differences in utilization were observed in comparisons of recently-constructed LL schools with unrenovated schoolyards (table 3). Although there were observable differences in other comparisons, the only statistically significant difference was among number of boys observed when comparing recent with older LL construction.

**Table 3:** Differences in school yard utilization by construction status for all periods, reported in number of children observed per number of children per 100 children enrolled in the school

	Old LL vs. Control		New LL vs. Control		New LL vs. Old LL	
	mean difference	p	mean difference	p	mean difference	p
**All Children**	2.4	.194	7.0	**.003	4.6	.056
**Boys**	0.8	.416	4.1	**.003	3.3	*.014
**Girls**	1.7	.087	2.9	**.006	1.2	.264

* p < .05 ** p < .01

We also report the *percentage *of children engaged in moderate to vigorous physical activity as a portion of all the children observed (tables 4 and 5). These data address a different question: whether children, once they are physically on a renovated schoolyard, are more likely to engage in physical activity than on unrenovated sites. There were no statistically significant differences observed between renovated and unrenovated schoolyards for any period for percentage of active children.

**Table 4:** Comparison of rate of moderately to physically active children observed on schoolyards at LL and control schools during different utilization periods, reported as percentage

	Mean rate, Learning Landscapes	Mean rate, unrenovated schoolyards	Mean difference	Standard error difference	p
**All periods**					
All children	42.2	40.4	1.7	3.3	.602
Boys	44.2	42.5	1.7	3.8	.646
Girls	39.7	38.5	1.2	3.3	.114
**Before School**					
All children	27.1	35.8	-8.6	4.1	.050
Boys	29.7	33.9	-4.2	6.0	.491
Girls	26.2	40.4	-14.1	9.8	.174
**Lunch Recess**					
All children	42.8	43.5	-0.7	2.6	.796
Boys	46.5	45.7	0.7	3.2	.841
Girls	38.7	40.1	-2.3	2.7	.402
**After School**					
All children	47.3	36.7	10.6	9.0	.242
Boys	48.0	40.6	6.3	10.5	.550
Girls	48.0	33.5	14.5	8.9	.111
**Weekends**					
All children	42.9	41.0	1.9	32.0	.956
Boys	44.2	42.9	1.3	31.4	.969
Girls	43.3	16.6	26.7	57.2	.665

**Table 5:** Differences in rate of moderately to physically active children observed for all periods, reported as a percent

	Old LL vs. control		New LL vs. control		New LL vs. old LL	
	mean difference	p	mean difference	p	mean difference	p
**All Children**	0.3	.993	3.2	.413	3.2	.409
**Boys**	-0.4	.928	3.7	.403	4.1	.341
**Girls**	1.1	.763	1.2	.776	0.7	.987

## Discussion

In light of the obesity epidemic and its associated health impacts in the US, parents, educators, and health researchers strive to discover which environmental interventions encourage physical activity among children. Among the ways that design strategies can contribute to addressing this problem, schoolyards can be constructed to encourage children to spend time outdoors and to actively utilize schoolyards. Evidence-based evaluation of the behavioral impacts of specific design initiatives is particularly important in a context in which funding is scarce. Such evidence is often not available or accessible to private- and public-sector policymakers [39].

While urban designers have discussed how specific designs of children's spaces might encourage healthy behavior, few studies have evaluated the effectiveness of these designs once they have been implemented. Outside of some evidence that the addition of color is related to increased utilization [32,40], little work addresses the impacts of specific design plans, either in terms of particular schoolyard components or as a broader design strategy. With the notable exception of a study from Cleveland [28], there has been little previous work on the impacts of renovation on schoolyard utilization. LL is a program which places high-quality recreational spaces into public schools serving Denver, including those with low-income populations. It was observed that LL schoolyards experienced significantly greater utilization for all observation periods over unrenovated spaces. When "optional" periods (before school, after school, and on weekends) are considered in isolation, greater utilization was generally observed for both boys and girls, with a few exceptions noted in the results.

There is evidence that boys are more likely than girls to exhibit greater utilization and more vigorous physical activity in both schoolyards in general [40] and on renovated playgrounds in particular [28]. While it remains unclear at what age weight-related differences in physical activity are apparent in girls [41], it is widely accepted that physical activity leads to benefits in physical health as well as social development [42,43]. In this study, greater utilization by girls was observed on LL sites before and after school and during lunch recess, but not on weekends. The reasons behind these findings merit additional scrutiny on several levels. Further analysis might reveal, for example, that specific features of the LL schoolyards encourage girls to use the sites in an active manner. Schoolyard use was generally much higher before school than it was after school. It seems plausible that girls in particular are more likely to utilize these spaces before and after school, in contrast to weekends, as a consequence of their family's perceived safety of having other children and school staff present. Low-income school districts that wish to implement extra-curricular programs to improve physical activity among their students, and among girls in particular, might benefit from research focused around this question. Future work might also investigate how age affects girls' utilization patterns.

In order to address the question of how renovation affected children's likelihood to engage in physical activity, we also examined the percentage of children who were moderately or vigorously active as a proportion of all children observed on the schoolyards. This measure is intended to estimate the way that a particular space influences the type of activity that children engage in; some play spaces might contain features that encourage children to participate in specific activities that are conducive to vigorous physical activity, for example. No statistically significant differences were observed in this measure between any of the comparison groups. It seems plausible that there are differences in how children utilize specific components of various play spaces, but this may not be observable at the scale of an entire school yard. A schoolyard zone containing slides, for example, may encourage more or less activity than another zone containing a jungle gym. Work to compare activity across schoolyard zones that contain different features might be a fruitful avenue for additional inquiry.

An additional feature of this research is the measurement of the impacts of the recency of schoolyard construction. Utilization of LL schoolyards constructed within the previous year was compared with schoolyards constructed between three and four years prior to the study period. Greater utilization was observed on LL sites that newer than one year than on the older LL sites, but the differences were not statistically significant, with the exception of the utilization measures among boys. If the differences observed in this work and evidence from other studies, such as Colabianchi et al.'s [28], are seen to provide evidence that schoolyard construction results in greater utilization and activity, schoolyard renovation itself may be a valid planning strategy for increasing utilization. Additional study is required to determine the duration of the impacts of renovation and whether these differences are the consequence of particular components of the renovated schoolyards.

### Study Limitations

While we believe that this work provides evidence of the impacts of schoolyard renovation, this study was designed as a case-control study, introducing the potential for confounding by unobserved or unconsidered third variables. A key question in study in study of physical activity and urban environments is how to implement changes that result in sustained changes in physical activity patterns. This work provides evidence that renovation of school yards results in an increase in physical activity, but it is not clear for how long this effect lasts. The evidence from this work also does not shed light on specific strategies for implementing sustained change in activity, a key focus for physical activity research and policy.

It might be possible that renovation only benefits children who are active to begin with, but who lack suitable facilities to practice physical activity in the deprived neighbourhoods examined here. While it is worthwhile to investigate strategies that reduce geographic and socioeconomic disparities in physical activity patterns, a key future goal is to uncover specific design strategies to encourage greater overall use among all children. Similarly, it would also be worthwhile to investigate specific design mechanisms responsible for increased use. Additional study of LL is presently considering these research questions in a longitudinal study design. While this study was originally designed to assess and compare utilization of LL and non-LL schoolyards, stratification into gender and time period resulted in a loss of statistical power. With additional data, these and other variables can be analyzed with greater statistical certainty.

It is possible that LL sites attracted greater use on account of their relatively recent date of construction, or perhaps some other factor associated therewith (such as general cleanliness, the state of the equipment, or the attention that was brought to the schoolyard by community involvement with the construction efforts and the associated publicity). Additional work comparing LL sites to other renovated schoolyards that were not constructed through the program is necessary to thread apart which of these factors is important. Finally, while statistically significant differences were observed, results were attenuated by the fact that there were only 264 observation scans across nine schools. The incorporation of more data would enable the examination of finer-scale distinctions between schoolyard use at different kinds of sites, and could enable more sophisticated analysis, by, for example, using a logistic regression analysis to model the impacts of particular site features.

## Conclusions

While encouraging physical activity among children is an important public health goal that may be addressed through a careful planning and design process, it is essential that specific strategies be explored and evaluated in order to determine how and to what extent these strategies encourage physical activity among children. This study provides evidence that schoolyard renovation from Learning Landscapes increases active utilization by school children during both mandatory and optional play periods, contributing to a nascent body of work [28,44] that suggests that such renovation may be an effective method to encouraging physical activity among children.

## Competing interests

The authors declare that they have no competing interests.

## Authors' contributions

BK, LB, and CN designed the study, obtained funding for, and managed the SOLPLAY data collection process. PA, LB, SL, EG, and BK contributed to the final version of the manuscript. PA and EG prepared the data for analysis. PA devised the current analysis, analyzed the data, and produced the initial draft of the manuscript. All authors read and approved the final manuscript.
